# Emergency cancer diagnosis in older adults: patterns, subgroups, and implications for health-care quality metrics

**DOI:** 10.1093/jncics/pkag008

**Published:** 2026-01-29

**Authors:** Sarah E Soppe, Sharon Peacock Hinton, Ellis C Dillon, Sandi L Pruitt, Georgios Lyratzopoulos, Matthew E Barclay, Megan A Mullins, Allison W Kurian, Nicholas Pettit, Matthew Thompson, Caroline A Thompson

**Affiliations:** Department of Epidemiology, Gillings School of Global Public Health, The University of North Carolina at Chapel Hill, Chapel Hill, NC, United States; Department of Epidemiology, Gillings School of Global Public Health, The University of North Carolina at Chapel Hill, Chapel Hill, NC, United States; Center on Aging, University of Connecticut, Farmington, CT, United States; Department of Social and Behavioral Sciences, Peter O’Donnell Jr School of Public Health, University of Texas Southwestern Medical Center, Dallas, TX, United States; Harold C. Simmons Comprehensive Cancer Center, University of Texas Southwestern Medical Center, Dallas, TX, United States; Epidemiology of Cancer Healthcare and Outcomes (ECHO), Department of Behavioural Science and Health, Institute of Epidemiology and Health Care (IECH), University College London, London, United Kingdom; Epidemiology of Cancer Healthcare and Outcomes (ECHO), Department of Behavioural Science and Health, Institute of Epidemiology and Health Care (IECH), University College London, London, United Kingdom; Harold C. Simmons Comprehensive Cancer Center, University of Texas Southwestern Medical Center, Dallas, TX, United States; Department of Health Economics, Systems, and Policy, Peter O’Donnell Jr School of Public Health, University of Texas Southwestern Medical Center, Dallas, TX, United States; Department of Epidemiology and Population Health, Stanford University School of Medicine, Stanford, CA, United States; Department of Medicine, Stanford University School of Medicine, Stanford, CA, United States; Department of Emergency Medicine, Indiana University School of Medicine, Indianapolis, IN, United States; Department of Family Medicine, University of Washington, Seattle, WA, United States; Department of Epidemiology, Gillings School of Global Public Health, The University of North Carolina at Chapel Hill, Chapel Hill, NC, United States; Lineberger Comprehensive Cancer Center, University of North Carolina at Chapel Hill, Chapel Hill, NC, United States

## Abstract

**Background:**

Cancer diagnosis originating in emergency departments (emergency presentation) contributes to poorer cancer survival and reflects aggressive disease and limited access to routine health care. This study characterized emergency presentations for a range of cancers and subclassified by whether patients were hospitalized after the emergency encounter, with the hypothesis that, compared with those hospitalized, patients not requiring hospitalization more specifically represent barriers to timely and adequate care.

**Methods:**

We analyzed Surveillance, Epidemiology, and End Results–Medicare data for patients aged 66 years and older diagnosed with 14 cancer types (2008-2017; *n* = 614 885). We described emergency presentation overall and demographic and clinical characteristics across subgroups using linear regression and assessed differences in health-care utilization before the emergency presentation classification window.

**Results:**

In total, 234 606 (38%) patients were classified as emergency presentations, with 187 439 (80%) hospitalized. Emergency presentations were more likely than nonemergency presentations to have prediagnostic emergency care (40%, 95% confidence interval [CI] = 40% to 40%) vs 30% (95% CI = 29% to 30%) and less likely to have nonemergency care for potential cancer symptoms (61%, 95% CI = 61% to 61%, vs 67%, 95% CI = 67% to 67%), with minimal variation between inpatient and outpatient emergency presentations. Compared with inpatient emergency presentations, outpatient emergency presentations were more often younger than 70 years old (24%, 95% CI = 23% to 24%, vs 19%, 95% CI = 19% to 19%), nonmetropolitan residents (25%, 95% CI = 24% to 25%, vs 12%, 95% CI = 12% to 12%), and had localized cancer (25%, 95% CI = 25% to 26%, vs 17%, 95% CI = 17% to 17%).

**Conclusions:**

More than one-third of older adult US cancer patients with these cancer types are diagnosed through emergency presentation, with most requiring hospitalization. Outpatient emergency presentations are more common among patients in rural areas with less advanced cancers, suggesting they may be an informative indicator of avoidable barriers to care less influenced by underlying health status.

## Introduction

Diagnostic delays worsen outcomes for cancer patients worldwide and may reflect missed opportunities to diagnose cancer through earlier ambulatory care.^1^^,^^2^ To pinpoint missed opportunities for diagnosis in administrative health-care data, diagnosis of cancer through care originating in the emergency department (ED) was recently proposed as a health-care quality metric.^2^ This measure of emergency presentation has been extensively examined outside the United States (US), often defined as cancer diagnosis following emergency hospital admission.^3^^,^^4^ Multiple studies identified emergency presentation as a predictor of poor survival independent of cancer stage.^3^^,^^4^ Yet, US estimates of emergency presentation prevalence and factors driving emergency presentation remain unreported for many cancer types.

Emergency presentations can be caused by a variety of circumstances, from rapid cancer progression with sudden severe symptoms to health-care access barriers resulting in reliance on the ED for primary care.^2^^,^^5-10^ Such barriers include extensive wait times in nonemergency settings, difficulty navigating the health-care system, poor care quality resulting in diagnostic errors, and other psychosocial and socioeconomic factors like lack of time and transportation for care.^11^^,^^12^ These factors are particularly relevant to the United States where the ED is frequently attended by patients with no regular health-care provider.^13^ Unlike aggressive cancer biology, barriers to care may be a more readily intervenable cause of emergency presentation. Better understanding of patients affected by emergency presentations, especially those that are avoidable, can translate to improved outcomes.

We previously reported the prevalence of emergency presentation of 4 common cancer types noting many patients had ED contact within 4 weeks before diagnosis.^5^ Here we report prevalence of emergency presentation across 14 cancer types examining subgroups of emergency presentation not previously presented. Many international studies define emergency presentation as emergency hospitalization before cancer diagnosis, often within the prior month,^3^^,^^4^ whereas most US studies consider any emergency care in the 30 days before diagnosis, combining patients hospitalized and discharged after emergency care.^2^^,^^5-7^ Here, we separate emergency presentations into patients diagnosed during or following an emergency hospitalization (“inpatient emergency presentations,” admission from the ED, or hospitalization of an emergency admission “type”), and patients diagnosed following discharge from the ED without hospitalization (“outpatient emergency presentations”). Because outpatient emergency presentations are able to be discharged, we hypothesized they would be less likely to have suddenly presented with severe symptoms indicative of unavoidable emergency presentation. We suspected this group may instead consist of patients presenting to the ED because of barriers to care related to socioeconomic disadvantage or access. To evaluate this hypothesis, we assessed whether outpatient emergency presentation patients have lower income or reside in rural neighborhoods with fewer prior nonemergency encounters for symptoms and higher overall ED use before cancer diagnosis. For additional international context, we contrasted emergency presentation prevalence among US older adults with similar metrics from England. To our knowledge, this is the first conceptualization of emergency presentations in this manner and the first United States-to-international comparison of emergency presentation prevalence.

## Methods

### Data source and study population

We used data from the Surveillance, Epidemiology, and End Results (SEER)–Medicare database.^14^ Patients were diagnosed from 2008 to 2017 with cancers of the colon, rectum, esophagus, stomach, liver and intrahepatic bile duct, pancreas, lung and bronchus, kidney and renal pelvis, urinary bladder, uterus, ovary, leukemia, non-Hodgkin lymphoma, and multiple myeloma. Breast and prostate cancers were excluded because of common detection through screening and low ED involvement.^5^ Because Medicare coverage typically begins at age 65 years and a year of fee-for-service claims was needed to classify prediagnostic care, we included patients aged 66 years and older with continuous Medicare Parts A and B and no Medicare Advantage enrollment in the year before diagnosis. We also excluded patients with prior cancers or diagnoses by autopsy or death certificate only.

### Classification of emergency presentation subgroups

**Figure 1.**
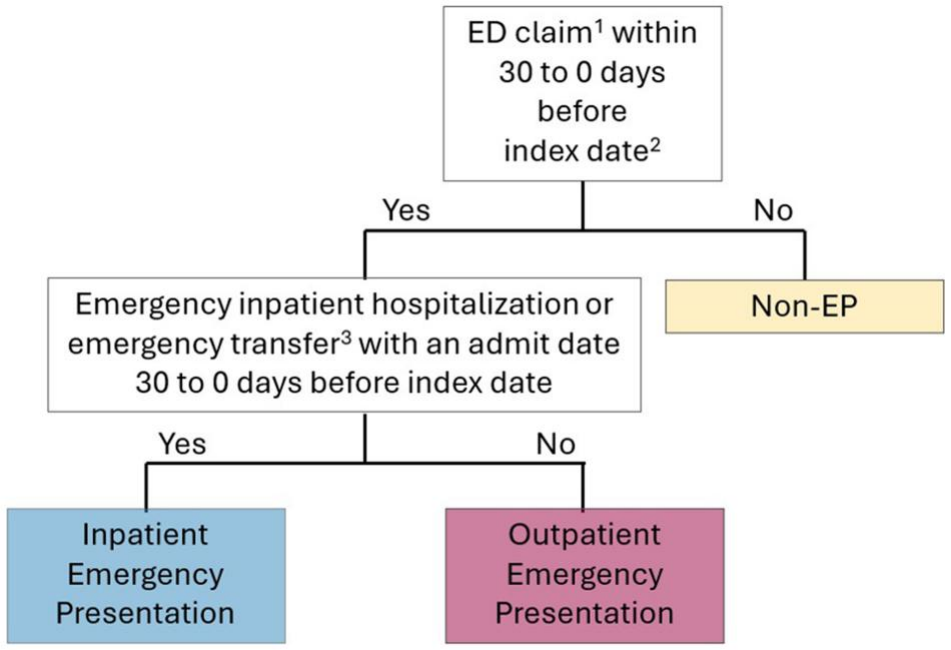
Algorithm for classifying inpatient and outpatient emergency presentation vs nonemergency presentation. ^1^ED-associated revenue code (0450-0459) OR a short-term acute care hospitalization (Medicare Provider Analysis and Review file) with an emergency admission type. ^2^Index date: Date of the earliest cancer-specific *International Classification of Diseases* code appearing on a claim in the Surveillance, Epidemiology, and End Results month of diagnosis (+/− 1 month). ^3^Emergency inpatient hospitalization or emergency transfer: The short-term acute care inpatient stay (Medicare Provider Analysis and Review file) has an ED-associated revenue code (0450-0459) OR the inpatient stay has an admission type of ED OR ED revenue code within 3 days before hospital admission. Abbreviations: ED = emergency department; EP = emergency presentation.

**Figure 2.**
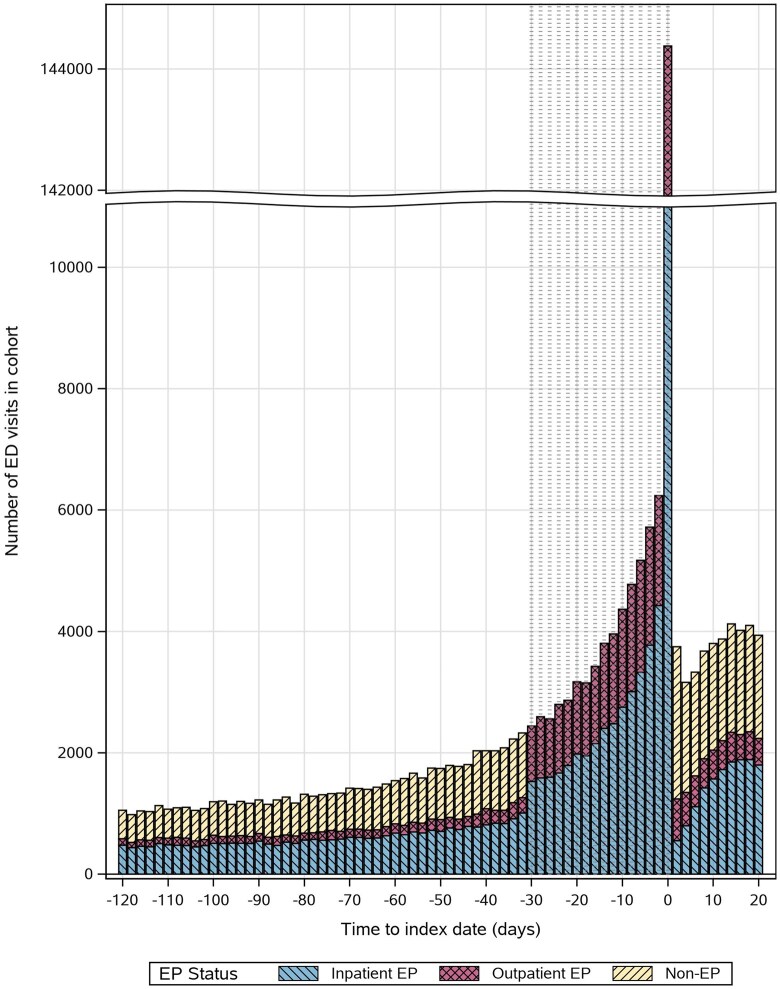
Frequency of all ED visits in the study cohort up to 120 days prediagnosis and 20 days postdiagnosis relative to the index date, colored by emergency presentation status. The lookback window for EP is shown in gray tick marks. Abbreviations: ED = emergency department; EP = emergency presentation.

Using a published algorithm,^15^^,^^16^ we derived the date of the earliest cancer-specific *International Classification of Diseases* (ICD) code in the SEER month of diagnosis (+/- 1 month) as the “index date” (Table S1). All patients with an emergency-associated claim in the 30 days before this date were classified as emergency presentations based on ED revenue codes (0450-0459) or hospitalizations with an emergency admission type, while all others were considered nonemergency presentations (see Figure 1). To explore adequacy of the 30-day lookback, we plotted the frequency of ED visits 120 days before the index date color-coded by emergency presentation status (see Figure 2). We subclassified emergency presentations based on their ED discharge disposition: inpatient emergency presentation if ED contact resulted in emergency hospitalization (see Figure 1) and outpatient emergency presentation if not.

### Patient sociodemographic and clinical characteristics

We obtained sociodemographic variables from SEER, including age, sex, race and ethnicity (Table S2), neighborhood-level socioeconomic status (SES),^17^ and county urbanicity based on US Department of Agriculture Rural Urban Continuum Codes.^18^ We also examined clinical characteristics, including tumor site, SEER summary stage, the Charlson Comorbidity Index,^19^ the Kim frailty index with preestablished cut points, and Medicaid dual eligibility from the Medicare files.^20^^,^^21^ To reflect clinical characteristics before cancer diagnosis, comorbidity and frailty indices were calculated using claims 90 to 365 days before the index date.

### English emergency presentation estimates

We used publicly available age-stratified National Health Service (NHS) summary statistics on routes to diagnosis, which use an algorithmic classification for different prediagnostic care scenarios.^3^ NHS emergency presentations include patients diagnosed via accident and emergency, emergency general practitioner referral, emergency transfer, or emergency admission or attendance, with most emergency presentations including hospitalization.^3^^,^^22^ We calculated the English emergency presentation proportion as the number of patients diagnosed via emergency presentation divided by the number of total cancer patients diagnosed during the years under study, comparing with both the US inpatient emergency presentation and total emergency presentation proportions.^23^ To improve comparability with our cohort, we excluded patients aged younger than 70 years and those with leukemia, lymphoma, and myeloma, for which NHS data were not publicly available, and collapsed the colon and rectum cancer groups. Differences in emergency presentation prevalence were assessed using tests of proportions (Table S3).

### Prediagnostic health-care utilization

We defined the *prediagnostic period* as the year before the index date, excluding the 30-day window used to classify emergency presentations. We quantified prediagnostic care including ED visits and non-ED visits for potential cancer symptoms by emergency presentation status. For the latter, we reviewed studies of symptoms for each cancer type (eg, abdominal bloating for colorectal cancer, chronic cough for lung cancer)^24-28^ (Table S4) and flagged claims with these ICD-9 and ICD-10 codes. To avoid mixing ED utilization with symptom-related care, we ignored symptom ICD codes on the same day as an ED visit.

### Statistical analysis

We reported the proportion of inpatient emergency presentations, outpatient emergency presentations, and nonemergency presentations overall and by stage and tumor site. We estimated prevalence differences for demographic and clinical characteristics described above using separate linear regression models with nonemergency presentations as the reference group,^29^^,^^30^ adjusting for age (modeled using 5-year age categories), SEER summary stage, sex, and tumor type. We also estimated prevalence differences for outpatient vs inpatient emergency presentations (excluding nonemergency presentations) using the same adjustment set. All statistical analyses were conducted in SAS v9.4 (SAS Institute, Cary, NC, USA).

## Results

**Figure 3.**
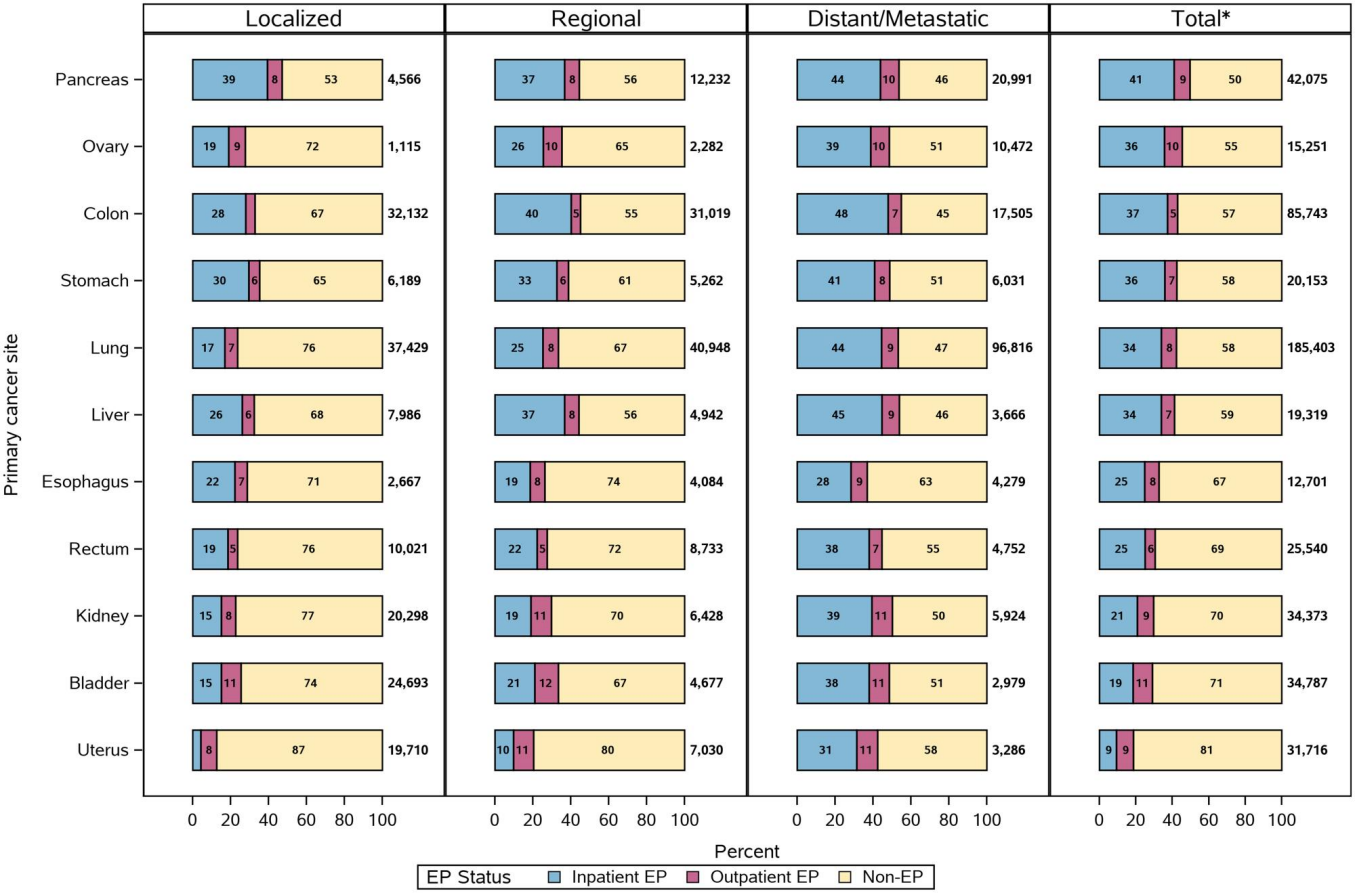
The proportion of cancers diagnosed as an inpatient and outpatient emergency presentation (EP) overall and by cancer site and stage across the 11 solid tumor sites, including from left to right inpatient EPs, outpatient EPs, and non-EPs. *Total includes unstaged cancers.

Of 614 885 included beneficiaries, 234 606 (38%) were diagnosed as emergency presentations, among whom 187 439 (80%) were hospitalized following emergency care (inpatient emergency presentations) (Table 1). Across the study population, the greatest frequency of ED visits occurred on the index date marking diagnosis. Increases in ED visits were observed as early as 70 days before diagnosis, with steeper increases during the 30-day lookback window defining emergency presentation (Figure 2). Emergency presentation prevalence was greatest for patients with cancers of the pancreas (50%), ovary (45%), and colon (43%) and lowest for those with kidney (30%), bladder (29%), and uterine cancer (19%) (Figure 3). Inpatient emergency presentations were more prevalent among those with later stage cancer (43% of metastatic vs 19% of localized), yet outpatient emergency presentations did not vary substantially by cancer type or stage (9% of metastatic vs 7% of localized).

**Table 1.** Patient characteristics by emergency presentation (EP status (inpatient EP, outpatient EP, non-EP)

Characteristics	All patients (*n* = 614 885) (100%)	Non-EP (*n* = 380 279) (62%)	Inpatient EP (*n* = 187 439) (30%)	Outpatient EP (*n* = 47 167) (8%)
	No. (col %)	No. (col %) [row %]	No. (col %) [row %]	No. (col %) [row %]
Sex				
Male	295 575 (48)	186 285 (49) [63]	86 953 (46) [29]	22 337 (47) [8]
Female	319 310 (52)	193 994 (51) [61]	100 486 (54) [32]	24 830 (53) [8]
Age, y				
66–70	144 613 (24)	97 850 (26) [68]	35 600 (19) [25]	11 163 (24) [8]
71–75	142 925 (23)	94 796 (25) [66]	37 388 (20) [26]	10 741 (23) [8]
76–80	128 586 (21)	80 914 (21) [63]	37 872 (20) [30]	9800 (21) [8]
81–85	105 595 (17)	61 343 (16) [58]	36 291 (19) [34]	7961 (17) [8]
86–90	64 865 (11)	33 217 (9) [51]	26 532 (14) [41]	5116 (11) [8]
91–95	23 704 (4)	10 370 (3) [44]	11 363 (6) [48]	1971 (4) [8]
Older than 95	4597 (<1)	1789 (<1) [39]	2393 (1) [52]	415 (<1) [9]
Race and ethnicity				
Non-Hispanic White	502 455 (82)	317 309 (83 [63]	146 526 (78) [29]	38 620 (82) [8]
Non-Hispanic Black	45 053 (7)	22 946 (6) [51]	18 529 (10) [41]	3578 (8) [8]
Hispanic	34 139 (6)	19 010 (5) [57]	12 278 (7) [36]	2851 (6) [8]
American Indian or Alaska Native	2404 (<1)	1465 (<1) [61]	664 (<1) [28]	275 (<1) [11]
East Asian American	14 394 (2)	9247 (2) [64]	4422 (2) [31]	725 (2) [5]
South Asian American	1877 (<1)	1210 (<1) [65]	541 (<1) [29]	126 (<1) [7]
Southeast Asian American	6980 (1)	3946 (1) [57]	2572 (1) [37]	462 (1) [7]
Other Asian American^a^	3362 (<1)	2142 (<1) [64]	1013 (<1) [30]	207 (<1) [6]
Native Hawaiian or other Pacific Islander	1437 (<1)	795 (<1) [55]	495 (<1) [34]	147 (<1) [10]
Mixed race, another race, or unknown race^b^	2784 (<1)	2209 (<1) [56]	399 (<1) [36]	176 (<1) [8]
Tumor site				
Bladder	34 787 (6)	24 628 (7) [71]	6461 (3) [19]	3698 (8) [11]
Colon	85 743 (14)	48 969 (13) [57]	32 133 (17) [38]	4641 (10) [5]
Esophagus	12 701 (2)	8542 (2) [67]	3170 (2) [25]	989 (2) [8]
Kidney	34 373 (6)	24 169 (6) [70]	7149 (4) [21]	3055 (7) [9]
Leukemia	35 495 (6)	22 889 (6) [65]	10 564 (6) [30]	2042 (4) [6]
Liver	19 319 (3)	11 352 (3) [59]	6546 (4) [34]	1421 (3) [7]
Lung	185 403 (30)	107 124 (28 [58]	62 918 (34) [34]	15 361 (33) [8]
Lymphoma (non-Hodgkin)	51 892 (8)	35 033 (9) [68]	13 222 (7) [26]	3637 (8) [7]
Myeloma	20 437 (3)	12 953 (3) [63]	5991 (3) [29]	1493 (3) [7]
Ovary	15 251 (3)	8330 (2) [55]	5445 (3) [36]	1476 (3) [10]
Pancreas	42 075 (7)	21 166 (6) [50]	17 265 (9) [41]	3644 (8) [9]
Rectum	25 540 (4)	17 733 (5) [69]	6379 (3) [25]	1428 (3) [6]
Stomach	20 153 (3)	11 599 (3) [58]	7236 (4) [36]	1318 (3) [7]
Uterus	31 716 (5)	25 792 (7) [81]	2960 (2) [9]	2964 (6) [10]
Summary stage				
Solid tumors				
Localized	166 806 (27)	123 547 (33) [74]	31 294 (17) [19]	11 965 (25) [7]
Regional	127 637 (21)	81 059 (21) [64]	37 060 (20) [29]	9518 (20) [8]
Distant, metastatic	176 701 (29)	84 653 (22) [48]	76 336 (41) [43]	15 712 (33) [9]
Unknown stage	35 917 (6)	20 145 (5) [56]	12 972 (7) [36]	2800 (6) [8]
Nonsolid tumors	107 824 (18)	70 875 (19) [66]	29 777 (16) [28]	7172 (15) [7]
Tumor grade				
Well differentiated	35 113 (6)	26 574 (7) [76]	6 263 (3) [18]	2276 (5) [7]
Moderately differentiated	126 748 (21)	85 193 (22) [67]	33 382 (18) [26]	8173 (17) [6]
Poorly differentiated	99 840 (16)	62 800 (17) [63]	29 066 (16) [29]	7974 (17) [8]
Undifferentiated	36 442 (6)	24 656 (7) [68]	8027 (4) [22]	3759 (8) [10]
Hemopoietic and lymphoid neoplasms	85 506 (14)	58 418 (15) [68]	21 339 (11) [25]	5749 (12) [7]
Unknown	231 236 (38)	122 638 (32) [53]	89 362 (48) [39]	19 236 (41) [8]
Charlson Comorbidity Index (excluding 90 days prior to index)				
0	208 069 (34)	136 571 (36) [66]	55 562 (30) [27]	15 936 (34) [8]
1	145 230 (24)	93 744 (25) [65]	40 109 (21) [28]	11 377 (24) [8]
2	99 765 (16)	61 261 (16) [61]	30 732 (16) [31]	7772 (17) [8]
≥3	161 821 (26)	88 703 (23) [55]	61 036 (33) [38]	12 082 (26) [8]
Kim Frailty Index (excluding 90 days prior to index)				
Robust	312 123 (51)	208 012 (55) [67]	80 924 (43) [26]	23 187 (49) [7]
Pre-frail	256 595 (42)	152 787 (40) [60)	83 458 (45) [33]	20 350 (43) [8]
Mildly frail	40 901 (7)	17 708 (5) [43]	19 934 (11) [49]	3259 (7) [8]
Moderate-to-severely frail	5266 (1)	1772 (<1) [34]	3123 (2) [59]	371 (1) [7]
Medicaid eligibility (any vs none)				
Full	88 394 (14)	43 003 (11) [49]	38 145 (20) [43]	7246 (15) [8]
Partial	21 906 (4)	11 784 (3) [54]	7743 (4) [35]	2379 (5) [11]
None	504 585 (82)	325 492 (86 [65]	141 551 (76) [65]	37 542 (80) [7]
Neighborhood socioeconomic status, percentile				
0-20th	95 983 (16)	53 254 (14) [56]	34 119 (18) [36]	8610 (18) [9]
20th-40th	120 831 (20)	72 108 (19) [60]	38 161 (20) [32]	10 562 (22) [9]
40th-60th	124 942 (20)	77 073 (20) [62]	37 964 (20) [30]	9905 (21) [8]
60th-80th	125 855 (21)	80 152 (21) [64]	36 853 (20) [29]	8850 (19) [7]
80th-100th	124 393 (20)	83 587 (22) [67]	33 194 (18) [27]	7612 (16) [6]
Unknown	22 881 (4)	14 105 (4) [62]	7148 (4) [31]	1628 (4) [7]
County urbanicity				
Metropolitan	523 317 (85)	322 843 (85 [62]	>164 835 (>88) [32]	>35 632 (>76) [7]
Urban, nonmetropolitan	81 433 (13)	50 966 (13) [63]	20 253 (11) [25]	10 214 (22) [13]
Rural	10 073 (2)	6423 (2) [64]	2340 (1) [23]	1310 (3) [13]
Unknown	62 (<1)	47 (<1) [76]	< 11 (<1) [<24]	< 11 (<1) [<24]

Abbreviation: EP = emergency presentation.

a Includes all other Asian nationalities not otherwise specified in STable 2.

b “Another” race includes all other race and ethnicities not otherwise specified.

**Figure 4.**
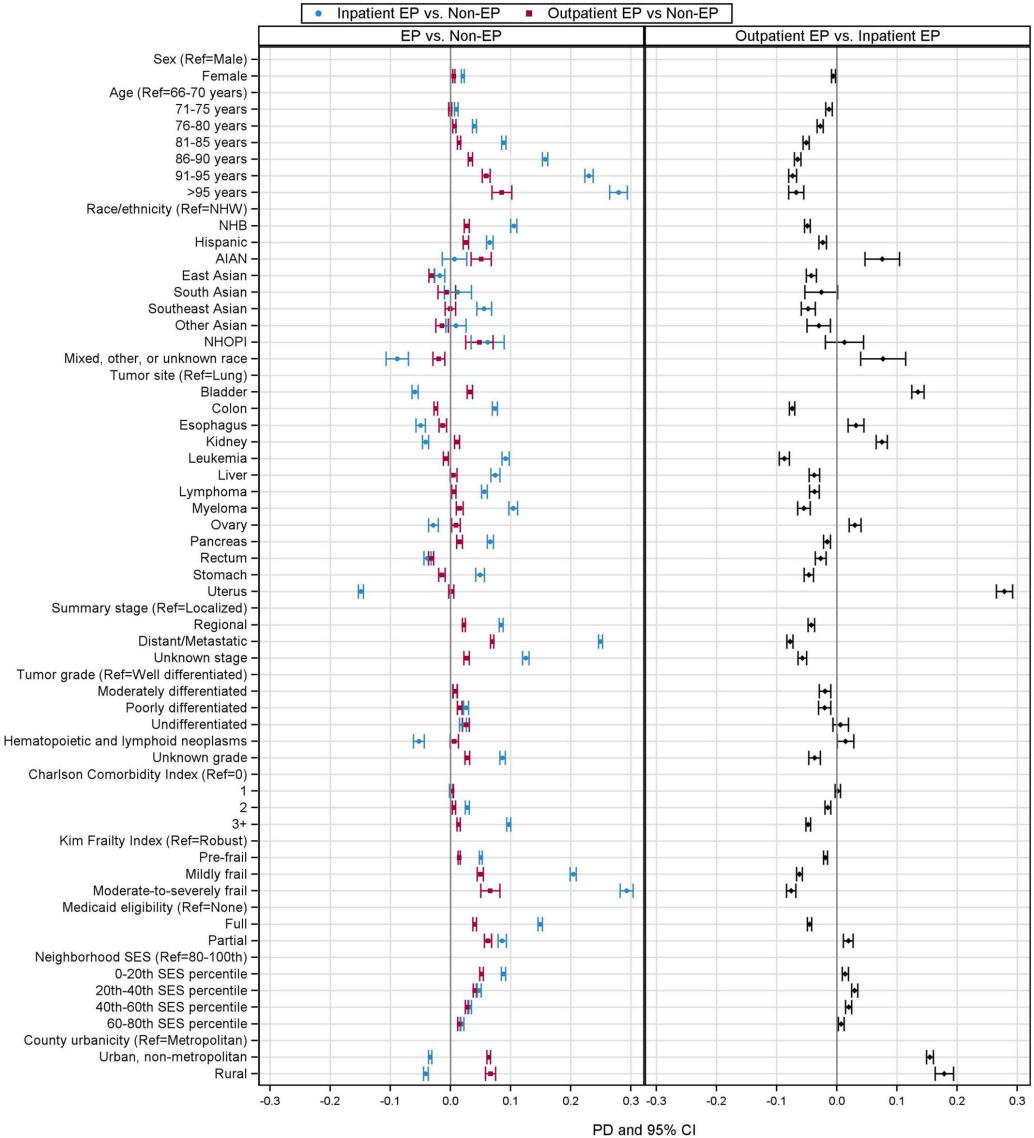
Adjusted prevalence differences of inpatient and outpatient emergency presentation (EP) (vs non-EP) and outpatient vs inpatient EP. Prevalence differences were generated using separate models for each characteristic additionally adjusted for age, sex, tumor site, and summary stage at diagnosis. Abbreviations: AIAN = American Indian or Alaska Native; CI = confidence interval; EP = emergency presentation; NHB = non-Hispanic Black; NHOPI = Native Hawaiian or other Pacific Islander; NWH = non-Hispanic White; PD = prevalence difference; ref = referent; SES = socioeconomic status.

Relative to nonemergency presentations, adjusted prevalence differences for inpatient emergency presentation were higher for patients who were older (prevalence difference = 28%, 95% confidence interval [CI] = 26% to 29%, for ages 95 years and older vs ages 66-70 years), frailer (prevalence difference = 29%, 95% CI = 28% to 30%, for moderate-to-severely frail vs robust), and more comorbid (prevalence difference = 10%, 95% CI = 9% to 10%, for Charlson Comorbidity Index ≥ 3 vs Charlson Comorbidity Index = 0) (Figure 4). Many of these factors were also associated with outpatient emergency presentations compared with nonemergency presentations, though with weaker prevalence differences. For example, outpatient emergency presentations were also more common among older patients (prevalence difference = 9%, 95% CI = 7% to 10%, for ages 95 years and older vs ages 66-70 years) and the highly frail (prevalence difference = 7%, 95% CI = 5% to 8%, for moderate-to-severely frail vs robust). Demographics associated with inpatient emergency presentation vs nonemergency presentation included specific race and ethnic groups (all vs non-Hispanic White): non-Hispanic Black (prevalence difference = 11%, 95% CI = 10% to 11%), Hispanic (prevalence difference = 7%, 95% CI = 6% to 7%), Southeast Asian (prevalence difference = 6%, 95% CI = 4% to 7%), Native Hawaiian or other Pacific Islander (prevalence difference = 6%, 95% CI = 3% to 9%); Medicaid eligibility (prevalence difference = 15%, 95% CI = 15% to 16%, for fully vs not eligible); and living in lower SES neighborhoods (prevalence difference = 9%, 95% CI = 8% to 9%, lowest vs highest quintile) (Figure 4). Non-Hispanic Black, Hispanic, and Native Hawaiian or other Pacific Islander race and ethnicity, dual eligibility, and lower neighborhood-level SES were also related to increased prevalence of outpatient emergency presentations (vs nonemergency presentations) but with associations equal to or less than those of inpatient emergency presentations. Only one race and ethnic group, American Indian or Alaska Native, was associated with outpatient emergency presentations (prevalence difference = 5%, 95% CI = 3% to 7%) and not inpatient emergency presentations. Relative to inpatient emergency presentations, outpatient emergency presentations were 8% (95% CI = 5% to 10%) higher among American Indian or Alaska Native (vs non-Hispanic White) individuals. Outpatient emergency presentations were also more common in nonmetropolitan counties compared with nonemergency presentations (prevalence difference = 6%, 95% CI = 6% to 7%, for nonmetropolitan urban, and prevalence difference = 7%, 95% CI =6% to 7%, for rural vs metropolitan counties). In models restricted to emergency presentations, these differences were substantial; rural emergency presentations had an 18% higher adjusted prevalence of outpatient emergency presentation (vs inpatient emergency presentation) compared with patients in metropolitan counties.

**Figure 5.**
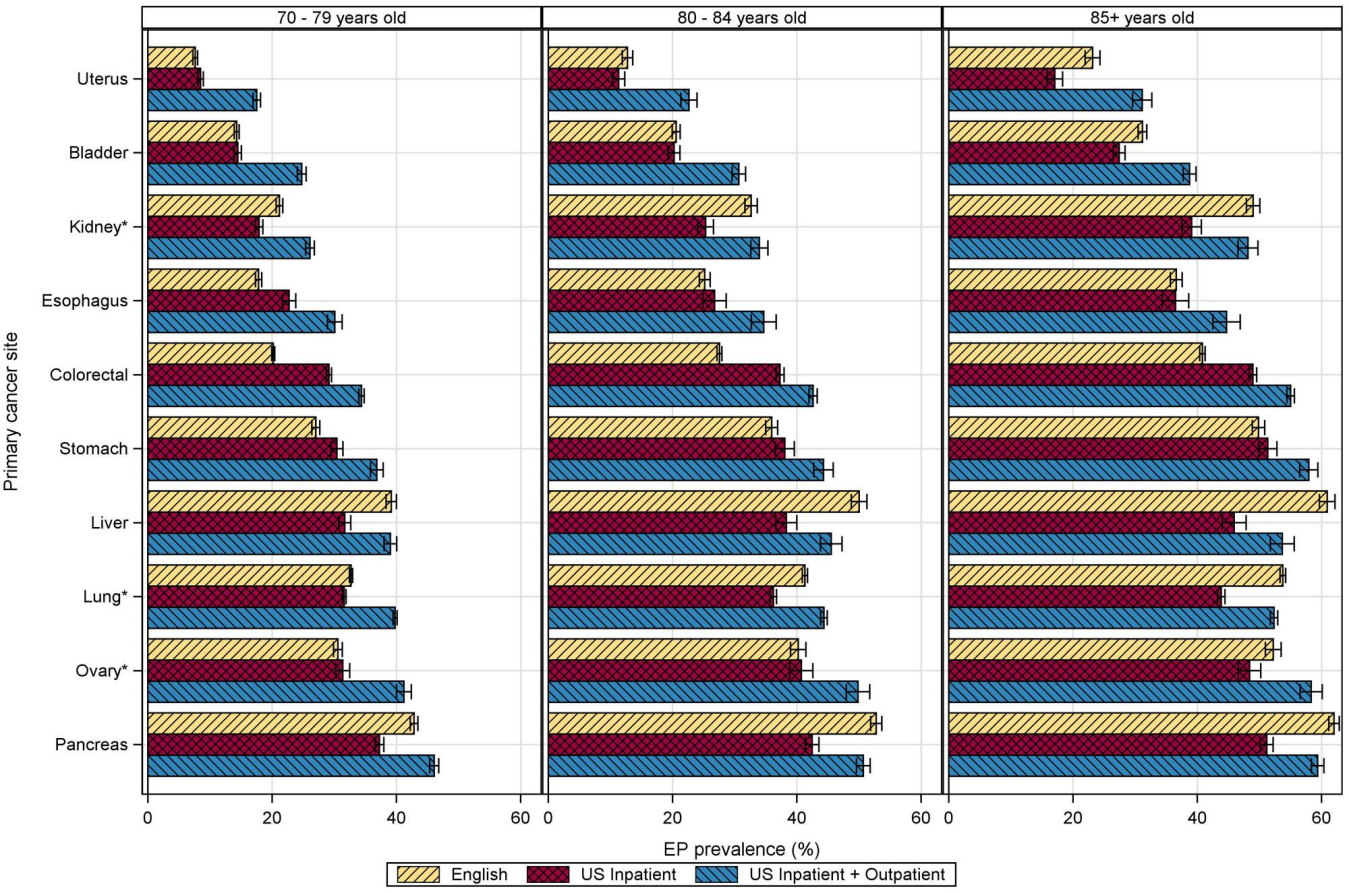
Proportion of emergency presentations in SEER-Medicare data compared with English estimates from National Health Service CancerData stratified by age group. Included age groups and cancer sites reflect the NHS data available.*Kidney cancers included renal pelvis in the SEER-Medicare data but not in the NHS data. Ovarian cancers included fallopian tube or primary peritoneal carcinomas, and lung cancers included tracheal cancers in the NHS data but not in the SEER-Medicare data. Abbreviations: EP = emergency presentation; NHS = National Health Service; SEER = Surveillance, Epidemiology, and End Results.

In the population of those aged 70 years and older with the 10 included cancer sites, US inpatient emergency presentations exceeded English emergency presentations for esophageal, colorectal, and stomach cancer, while English emergency presentations exceeded US inpatient emergency presentations for kidney, liver, lung, and pancreatic cancers (Figure 5). English emergency presentation proportions were similar to the US inpatient emergency presentation proportions for uterus, bladder, and ovarian cancer, aside from the oldest age group (85 years and older), among which English emergency presentations were greater. English emergency presentations exceeded the total US emergency presentation estimate of inpatient and outpatient emergency presentations for liver and pancreatic cancer.

**Figure 6.**
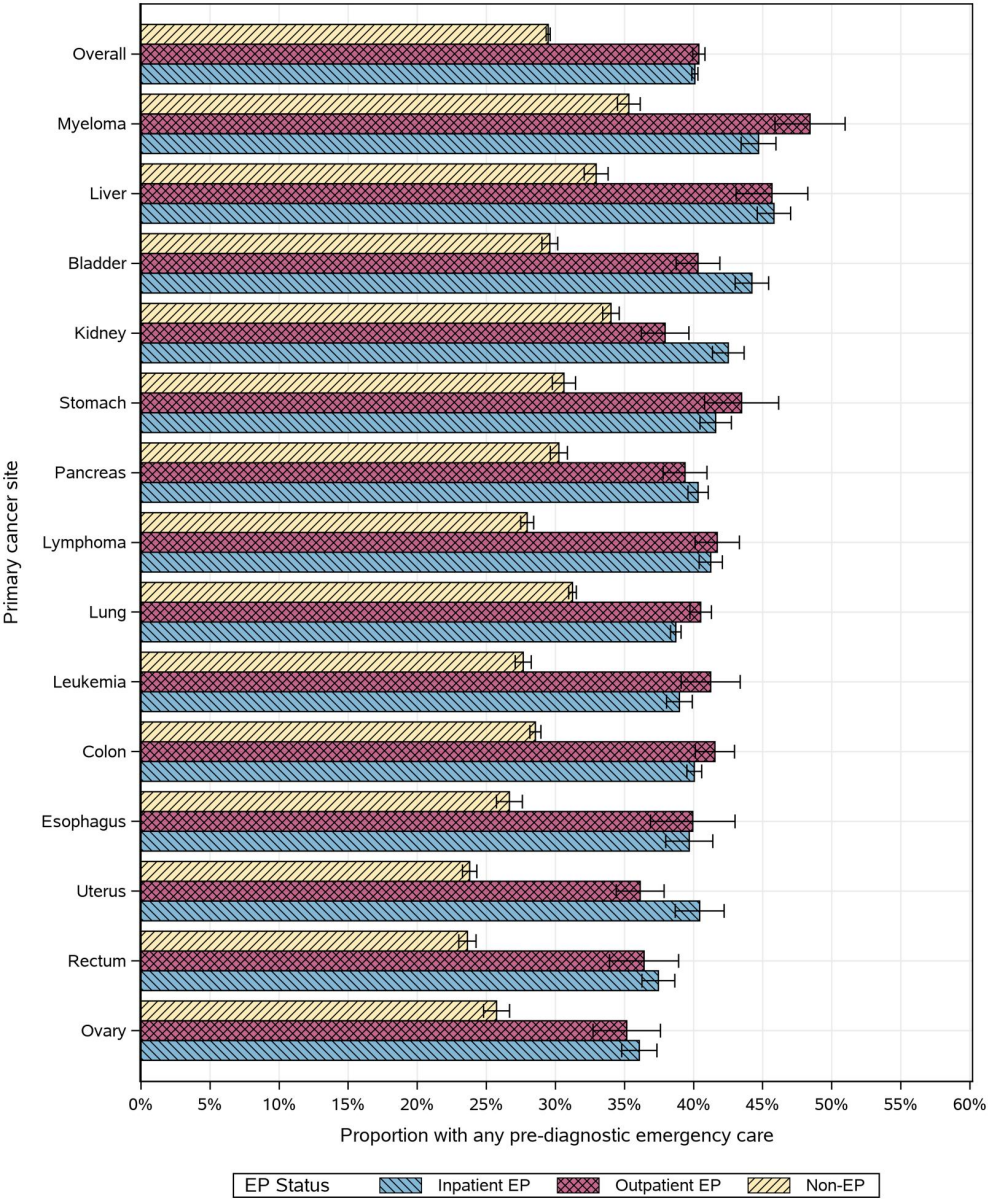
Proportion of patients with any emergency care in the prediagnostic period before the emergency presentation (EP) window (ie, excluding the 30 days before the index), stratified by cancer site and EP status.

Fewer nonemergency presentations had prediagnostic ED visits (30%, 95% CI = 29% to 30%), but this proportion did not vary overall between inpatient (40%, 95% CI = 40% to 40%) and outpatient (40%, 95% CI = 40% to 41%) emergency presentations (Figure 6). Stratified by cancer type, more patients had prediagnostic ED utilization among inpatient emergency presentations than outpatient emergency presentations for uterine, kidney, and bladder cancer. Alternatively, more outpatient emergency presentation patients had prediagnostic ED visits than inpatient emergency presentations for those with lung cancer and myeloma.

**Figure 7.**
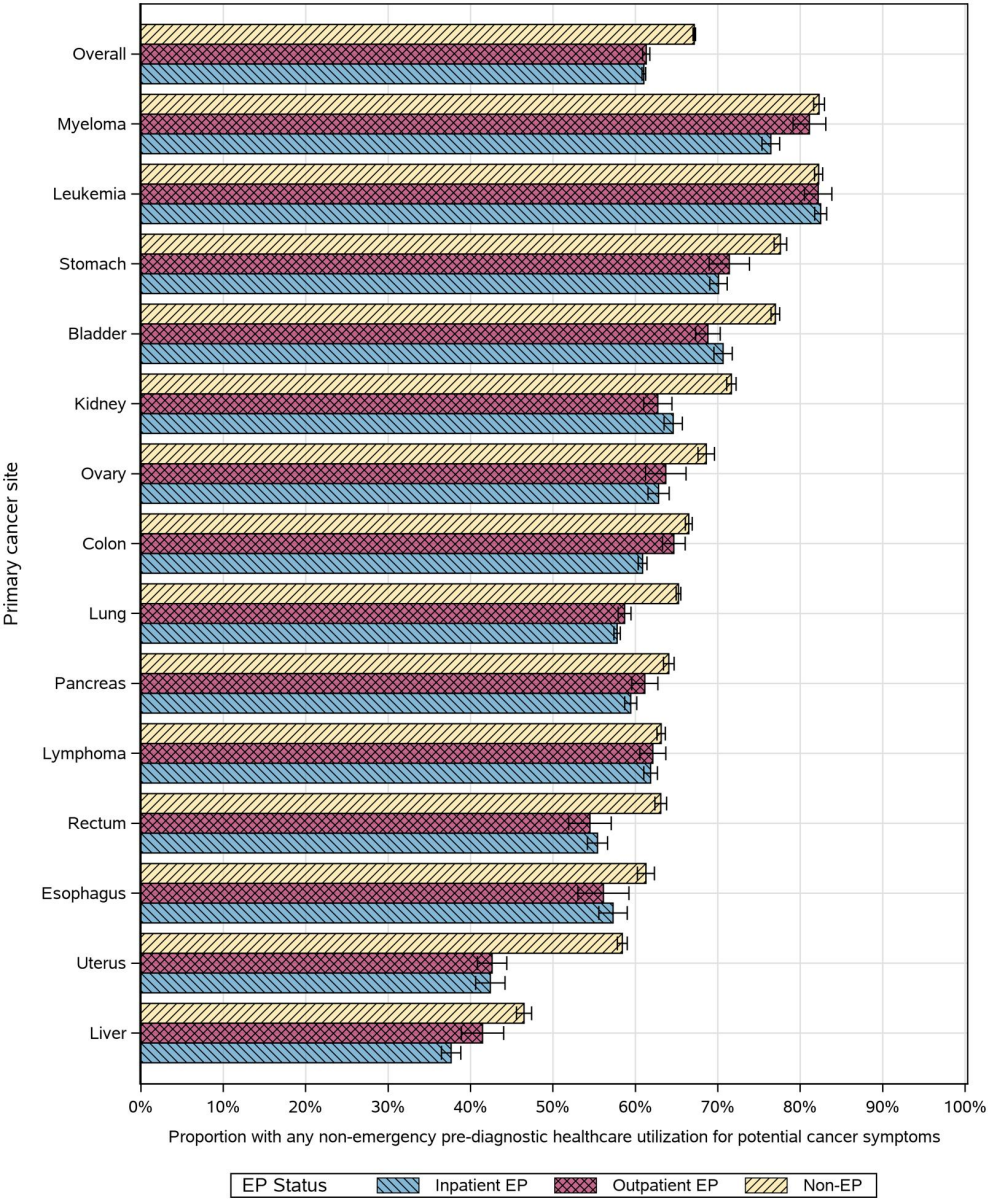
Proportion of patients with any nonemergency health-care utilization for site-specific potential cancer symptoms in the prediagnostic period before the emergency presentation (EP) window (ie, excluding the 30 days before the index), stratified by cancer site and EP status.

Patients with nonemergency prediagnostic care for potential cancer symptoms before the emergency presentation lookback window were slightly more prevalent among nonemergency presentations (67%, 95% CI = 67% to 67%) compared with emergency presentations, also with little variation between inpatient (61%, 95% CI = 61% to 61%) and outpatient (61%, 95% CI = 61% to 62%) emergency presentations (Figure 7). By cancer site, this care was slightly more common for outpatient vs inpatient emergency presentations among patients with myeloma, colon, or liver cancer; no cancer type had a statistically greater prevalence of nonemergency care among inpatient emergency presentations than outpatient emergency presentations (Figure 7).

## Discussion

We report emergency presentation prevalence across a range of cancer types, including many not previously reported in US populations, among Medicare beneficiaries reflecting most US adults aged older than 65 years. More than 1 in 3 patients with pancreatic, ovarian, colon, stomach, myeloma, lymphoma, lung, liver, and esophageal cancers were diagnosed through an emergency presentation route. These cancer sites have poor survival and may be overrepresented among emergency presentations because of aggressive disease or broad, nonspecific symptom profiles complicating timely diagnosis in routine care.^24^^,^^31^ In contrast, sites with lower emergency presentation prevalence in our study (eg, uterus and bladder) tend to have more specific and alarming symptoms, such as postmenopausal bleeding or hematuria, which may prompt more straightforward diagnostic evaluation.^24^^,^^32^^,^^33^ Emergency presentation prevalence in this study was nearly double that of recent US estimates for colorectal and lung cancer using electronic health records.^2^^,^^6^ This may reflect the older age of our cohort and more complete capture of emergency care in Medicare claims compared with electronic health records, which often miss care outside a given health-care system.^34^ Consistent with prior findings,^10^ emergency presentation was more prevalent among patients with poor underlying health and advanced cancer, as well as barriers to care including lower neighborhood SES and geographic isolation.

Unlike prior studies, we examined emergency presentations stratified by care pathway following ED evaluation. Patients diagnosed as outpatient emergency presentations were a distinct group with less clinical acuity and more rurality than inpatient emergency presentations. Although existing emergency presentation literature primarily emphasizes factors driving unavoidable emergency care, such as older age, greater comorbidity, and frailty, and more advanced stage, we observed that these associations were substantially weaker for outpatient emergency presentations. Even in this insured population, outpatient emergency presentations were also more likely than nonemergency presentations to live in disadvantaged neighborhoods and rural counties, areas often affected by provider shortages and limited health-care access.^35^ Although inpatient emergency presentations also appeared driven partly by access barriers, outpatient emergency presentations did not share the same associations with later stage or poorer clinical condition as inpatient emergency presentations, indicating outpatient emergency presentations may more specifically reflect lack of access to a usual source of quality care rather than clinical acuity. Combining US inpatient and outpatient emergency presentations may obscure important heterogeneity in the drivers of emergency presentation. Specifically examining outpatient emergency presentations may help identify modifiable missed opportunities for earlier diagnosis, as these may more strongly reflect barriers to care rather than severe disease.

Although the English emergency presentation estimates were produced by a different algorithm than used here, reflecting different data sources and underlying health-care systems, they may conceptually be most comparable to US inpatient emergency presentations because most English emergency presentations include a hospital admission.^22^ The English algorithm uses a 28-day prediagnostic window rather than 30 days as used here and in most US studies, though this has been shown to minimally impact results.^3^^,^^4^ The proportion of patients diagnosed through emergency presentation in England was similar to our estimates for many cancer sites, though with some variability for gastrointestinal cancers. Variation in emergency presentation estimates across countries may generate hypotheses about how differences in earlier care could influence emergency presentation. For instance, the greater prevalence of US inpatient emergency presentation for colorectal cancers relative to England may reflect differences in screening uptake and frequency.^36^ However, dissimilarities in data sources, algorithms, and health-care systems should be kept in mind; many English emergency presentations are classified based on emergency general practitioner referrals not explicitly quantified here,^22^ while the lack of a usual source of primary care may not contribute to English emergency presentation as substantially.

As previously reported,^5^^,^^9^^,^^10^ our findings indicate several racial and ethnic groups are disproportionately affected by emergency presentations. We report for the first time that non-Hispanic Black, Hispanic, Southeast Asian, and Native Hawaiian or other Pacific Islander patients had stronger associations with inpatient emergency presentation than outpatient emergency presentation, which may reflect greater comorbidity or cancer aggressiveness resulting from the accumulation of systemic health barriers.^37^ Interestingly, American Indian or Alaska Native patients did not follow this pattern, with higher prevalence of outpatient emergency presentations than other groups even after adjusting for age, sex, stage, and cancer site. Some American Indian or Alaska Native patients may receive emergency services through the Indian Health Service, which lacks specialized cancer care and may be more likely to reside on rural reservation lands, factors that could contribute to outpatient-only ED care.^38^ These patterns and subsequent consequences of outpatient emergency presentations on outcomes in these populations warrant further investigation.

Patients diagnosed as emergency presentations were more likely to have ED visits in the year before diagnosis, suggesting greater reliance on the ED as a usual source of care. We hypothesized prediagnostic ED use would be even higher among patients diagnosed through outpatient emergency presentations than inpatient emergency presentations; yet, aside from a few site-specific exceptions, both groups were equally likely to have prediagnostic emergency care. As an older, frailer, and more comorbid group, inpatient emergency presentations may have greater need for emergency care, resulting in high ED utilization regardless of access barriers. Although the proportion receiving prediagnostic care for potential cancer symptoms outside the ED was somewhat lower for emergency presentations than nonemergency presentations, this difference was less than 5 percentage points, challenging the notion that emergency presentation patients simply did not seek other care for symptoms before presenting to the ED. We also hypothesized outpatient emergency presentations would have less prediagnostic care for cancer symptoms than inpatient emergency presentations, though the proportions were ultimately similar across both groups. Nonetheless, we could not assess whether quality of prediagnostic care differed between subgroups, which could partly explain the reliance of patients with outpatient emergency presentation on the ED, even in the absence of disease severity requiring hospitalization.

Our findings assume emergency care in the 30 days before the index date (a convention used in many prior studies) was related to the soon-to-be diagnosed cancer rather than unrelated conditions.^2^^,^^4-7^ To assess the reliability of this window, we examined the density of ED visits over time, which increased as early as 70 days before the index. This suggests some earlier ED visits may relate to cancer symptoms; the best lookback window for measuring emergency presentation should be evaluated further. Strengths of this study included use of Medicare data well-suited for identifying emergency presentations because of continuity of coverage after enrollment and inclusion of care across different hospital systems. Although some emergency care may have been billed to secondary insurance and not captured here, we expect this was minimal. Importantly, our findings are less generalizable to younger patients and individuals without continuous health insurance, who may have more access barriers and rely more heavily on the ED for routine care needs. Additionally, our study underrepresents Medicare Advantage beneficiaries, an increasingly large segment of the Medicare population who may have better access to primary care and care coordination services.^39^ Future work should assess whether emergency presentation patterns differ in these groups and examine how differences in care continuity affect emergency presentations and missed diagnostic opportunities.

Our results provide comprehensive estimates of emergency presentation among US older adults with previously understudied cancer types and suggest the presence of meaningful subgroups within emergency presentation, revealing important heterogeneity that may be obscured when these subgroups are combined. Stratifying emergency presentations by whether patients were hospitalized after emergency care may help pinpoint drivers of avoidable emergency presentations, with outpatient emergency presentations serving as more specific indicators of care quality less affected by unmodifiable factors like underlying health status and cancer aggressiveness.^2^^,^^40^ Targeted efforts are needed to strengthen primary care access, care continuity, and early diagnostic evaluation, particularly in rural and underserved areas. Future studies should examine quality and timeliness of prediagnostic care in these subgroups and develop strategies to reduce missed diagnostic opportunities, with a particular focus on outpatient emergency presentations.

## Supplementary Material

pkag008_Supplementary_Data

## Data Availability

The data underlying this article cannot be shared because of institutional review board constraints around patient privacy. SEER-Medicare data can be accessed through an application to the National Cancer Institute with a SEER-Medicare Data Use Agreement and institutional review board approval. Analytic code used in this analysis will be made available online.
