# Supplementary material for: Emergency cancer diagnosis in older adults: patterns, subgroups, and implications for health-care quality metrics
Source: JNCI Cancer Spectr. 2026 Jan 29;10(2):pkag008. doi: 10.1093/jncics/pkag008 (PMC12953026; doi:10.1093/jncics/pkag008)
Supplement: pkag008_Supplementary_Data [file pkag008_supplementary_data.docx]

**Supplementary Material**

Table S1. International Classification of Diseases codes used to identify the index date as the day of the earliest cancer site-specific Medicare claim across 14 cancer sites.

| **Cancer Site** | **SEER-WHO recode** | **ICD-9** | **ICD-10** |
| --- | --- | --- | --- |
| **bladder** | 29010 | 188.0-188.9 | C67 |
| **colon** | 21041-21049 | 153.0-153.9 | C18 |
| **esophagus** | 21010 | 150.0-150.9 | C15, C49A1 |
| **kidney and renal pelvis** | 29020 | 189.0-189.1, 209.24 | C64.1-C64.2, C64.9, C65.1-C65.2, C65.9 |
| **leukemia** | 35011-35013, 35021-35023, 35031, 35041, 35043 | 202.4-208.92 | C91-C95 |
| **liver and intrahepatic bile duct** | 21071-21072 | 155.0-155.2 | C22 |
| **lung and bronchus** | 22030 | 162.2-162.9 | C34 |
| **lymphoma**  **(Non-Hodgkin's)** | 33041-33042 | 200.0-200.88, 202.0-202.98 | C82-C88, C96.0-C96.29, C96.5-C96.9, C96Z |
| **myeloma** | 34000 | 203.0-203.02 | C90 |
| **ovary** | 27040 | 183.0 | C56 |
| **pancreas** | 21100 | 157.0-157.9 | C25 |
| **rectum** | 21051-21052 | 154.0-154.3, 154.8 | C19-C20 |
| **stomach** | 21020 | 151.0-151.9, 209.23 | C16, C49A2 |
| **corpus and uterus** | 27020-27030 | 179, 182.0-182.1, 182.8 | C54-C55 |

Table S2. Nationalities included in the race/ethnicity categories derived from the SEER cancer registry.

| **Race and ethnicity** | **Included nationalities** |
| --- | --- |
| **East Asian American** | Chinese, Korean, Japanese, or Taiwanese |
| **South Asian American** | Bangladeshi, Bhutanese, Indian, Maldivian, Nepalese, Pakistani, and Sri Lankan |
| **Southeast Asian American** | Cambodian, Indonesian, Laotian, Malaysian, Singaporean, Thai, Philippine, and Vietnamese |
| **Native Hawaiian or other Pacific Islander** | Polynesian, Micronesian, and Melanesian |

Table S3. Proportion of Emergency Presentations in SEER-Medicare data compared to English estimates from National Health Service (NHS) CancerData stratified by age group, including Prevalence Differences with English data as the reference group. P-values were calculated from a test of proportions. Included age groups and cancer sites reflect the NHS data available.*

| **Cancer site** | **Age group (years)** | **English Estimate**  **(95% CI)** | **US Inpatient**  **Estimate**  **(95% CI)** | **Prevalence Difference (%)**  **(REF: English)**  **(95% CI)** | **p-value** | **US Inpatient + Outpatient Estimate**  **(95% CI)** | **Prevalence**  **Difference**  **(%)**  **(REF: English)**  **(95% CI)** | **p-value** |
| --- | --- | --- | --- | --- | --- | --- | --- | --- |
| Bladder | 70 – 79 | 14.3  (13.9, 14.7) | 14.5  (13.9, 15.0) | 0.2  (-0.9, 0.5) | 0.61 | 24.8  (24.1, 25.5) | -10.5  (-11.3, -9.7) | <0.01 |
|  | 80 – 84 | 20.6  (19.9, 21.2) | 20.2  (19.3, 21.2) | 0.3  (-0.8, 1.4) | 0.57 | 30.7  (29.6, 31.7) | -10.1  (-11.3,  -8.8) | <0.01 |
|  | 85+ | 31.2  (30.5, 31.9) | 27.4  (26.5, 28.4) | 3.7  (2.6, 4.9) | <0.01 | 38.7  (37.7, 39.8) | -7.6  (-8.8, -6.3) | <0.01 |
| Colorectal | 70 – 79 | 20.2  (19.9, 20.4) | 29.1  (28.7,  29.6) | -9.0  (-9.5, -8.5) | <0.01 | 34.4  (33.9, 34.8) | -14.2  (-14.7, -13.7) | <0.01 |
|  | 80 – 84 | 27.5  (27.1, 27.9) | 37.3  (36.6,  37.9) | -9.7  (-10.5, -9.0) | <0.01 | 42.6  (41.9, 43.3) | -15.1  (-15.8, -14.3) | <0.01 |
|  | 85+ | 40.8  (40.4, 41.2) | 48.9  (48.3,  49.5) | -8.1  (-8.9, -7.4) | <0.01 | 55.0  (54.4, 55.6) | -14.2  (-14.9, -13.4) | <0.01 |
| Esophagus | 70 – 79 | 17.8  (17.3, 18.3) | 22.7  (21.6, 23.8) | -4.9  (-6.1, -3.7) | <0.01 | 30.1  (28.9, 31.3) | -12.3  (-13.5, -11.0) | <0.01 |
|  | 80 – 84 | 25.2  (24.3, 26.0) | 26.8  (24.9, 28.6) | -1.6  (-3.6, 0.5) | 0.13 | 34.7  (32.7, 36.7) | -9.5  (-11.7, -7.3) | <0.01 |
|  | 85+ | 36.6  (35.7, 37.5) | 36.5  (34.4,  38.6) | 0.1  (-2.2, 2.4) | 0.92 | 44.7  (42.5, 46.9) | -8.1  (-10.5, -5.7) | <0.01 |
| Kidney | 70 – 79 | 21.2  (20.6, 21.7) | 17.9  (17.3, 18.5) | 3.3  (2.5, 4.1) | <0.01 | 26.1  (25.4, 26.8) | -4.9  (-5.8, -4.0) | <0.01 |
|  | 80 – 84 | 32.6  (31.6, 33.6) | 25.3  (24.1, 26.6) | 7.3  (5.7, 8.9) | <0.01 | 34.0  (32.6, 35.3) | -1.3  (-3.0, 0.4) | 0.12 |
|  | 85+ | 49.0  (47.9, 50.1) | 39.1  (37.5, 40.6) | 9.9  (8.0, 11.8) | <0.01 | 48.1  (46.5, 49.7) | 0.8  (-1.1, 2.8) | 0.40 |
| Liver | 70 – 79 | 39.2  (38.3, 40.0) | 31.7  (30.7, 32.6) | 7.5  (6.2, 8.8) | <0.01 | 39.0  (38.0, 40.0) | 0.1  (-1.2, 1.4) | 0.83 |
|  | 80 – 84 | 50.0  (48.8, 51.3) | 38.3  (36.6, 40.0) | 11.7  (9.6, 13.8) | <0.01 | 45.5  (43.8, 47.3) | 4.5  (2.3, 6.6) | <0.01 |
|  | 85+ | 60.9  (59.6, 62.1) | 45.9  (44.0, 47.8) | 15.0  (12.7, 17.3) | <0.01 | 53.7  (51.7, 55.6) | 7.2  (4.9, 9.5) | <0.01 |
| Lung | 70 – 79 | 32.7  (32.4, 32.9) | 31.6  (31.3, 31.9) | 1.1  (0.7, 1.5) | <0.01 | 39.8 (39.5, 40.1) | -7.1  (-7.5, -6.7) | <0.01 |
|  | 80 – 84 | 41.3  (40.9, 41.7) | 36.2  (35.7, 36.7) | 5.1  (4.4, 5.7) | <0.01 | 44.3 (43.8, 44.9) | -3.1  (-3.7, -2.4) | <0.01 |
|  | 85+ | 53.8  (53.3, 54.2) | 43.8  (43.2, 44.4) | 9.9  (9.2, 10.7) | <0.01 | 52.3 (51.7, 55.6) | 1.4  (0.7, 2.2) | <0.01 |
| Ovary | 70 – 79 | 30.6  (29.9, 31.3) | 31.4  (30.2, 32.5) | -0.8  (-2.1, 0.5) | 0.25 | 41.2 (40.0, 42.4) | -10.6  (-12.0, -9.2) | <0.01 |
|  | 80 – 84 | 40.2  (39.0, 41.4) | 40.7  (38.9, 42.6) | -0.5  (-2.7, 1.7) | 0.66 | 49.8 (48.0, 51.7) | -9.6  (-11.9, -7.4) | <0.01 |
|  | 85+ | 52.2  (50.9, 53.5) | 48.4  (46.6, 50.2) | 3.8  (1.6, 6.0) | <0.01 | 58.3 (56.6, 60.1) | -6.1  (-8.3, -3.9) | <0.01 |
| Pancreas | 70 – 79 | 42.8  (42.2, 43.4) | 37.3  (36.5, 38.0) | 5.6  (4.6, 6.5) | <0.01 | 46.1 (45.3, 46.8) | -3.2  (-4.2, -2.3) | <0.01 |
|  | 80 – 84 | 52.8  (51.9, 53.7) | 42.4  (41.4, 43.5) | 10.3  (8.9, 11.7) | <0.01 | 50.7 (49.6, 51.8) | 2.1  (0.7, 3.5) | <0.01 |
|  | 85+ | 62.0 (61.1, 62.8) | 51.2  (50.1, 52.2) | 10.8  (9.5, 12.1) | <0.01 | 59.4 (58.3, 60.4) | 2.6  (1.3, 3.9) | <0.01 |
| Stomach | 70 – 79 | 27.0  (26.4, 27.7) | 30.4  (29.4, 31.4) | -3.4  (-4.6, -2.2) | <0.01 | 36.8 (35.8, 37.9) | -9.8  (-11.0,  -8.6) | <0.01 |
|  | 80 – 84 | 35.9  (35.0, 36.9) | 38.0  (36.5, 39.6) | -2.1  (-3.9, -0.3) | 0.02 | 44.3 (42.7, 45.8) | -8.3  (-10.2, -6.5) | <0.01 |
|  | 85+ | 49.8  (48.8, 50.8) | 51.3  (49.9, 52.8) | -1.5  (-3.3, 0.3) | 0.10 | 57.9 (56.5, 59.4) | -8.1  (-9.9, -6.3) | <0.01 |
| Uterus | 70 – 79 | 7.6  (7.3, 8.0) | 8.5  (8.0, 8.9) | -0.9  (-1.4, -0.3) | <0.01 | 17.5 (16.9, 18.1) | -9.9  (-10.6,  -9.2) | <0.01 |
|  | 80 – 84 | 12.7  (11.9, 13.6) | 11.3  (10.3, 12.3) | 0.3  (-0.8, 1.5) | 0.57 | 22.6 (21.3, 23.9) | -9.9  (-11.4, -8.3) | <0.01 |
|  | 85+ | 23.1  (21.9, 24.3) | 17.1  (15.8, 18.3) | 6.1  (4.3, 7.8) | <0.01 | 31.1 (29.6, 32.7) | -8.0  (-9.9, -6.0) | <0.01 |

* Kidney cancers included renal pelvis in the SEER-Medicare data but not in the NHS data. Ovarian cancers included fallopian tube or primary peritoneal carcinomas and lung cancers included tracheal cancers in the NHS data but not in the SEER-Medicare data.

Table S4. Billed symptoms used to assess healthcare utilization for potential cancer symptoms in the pre-diagnosis period.

| **Cancer Site** | **Symptom Categories** | **Source(s**)* |
| --- | --- | --- |
| Bladder | Abdominal pain | Koo et al, 2018 |
|  | Abdominal tenderness | Koo et al, 2018 |
|  | Anemia | Moore et al, 2021 |
|  | Back pain | ACS |
|  | Bone pain | ACS |
|  | Constipation | Koo et al, 2018 |
|  | Dysuria | Koo et al, 2018 |
|  | Increased frequency of urination | Moore et al, 2021 |
|  | Invisible hematuria | Koo et al, 2018 |
|  | Jaundice | Koo et al, 2018 |
|  | Malaise | ACS |
|  | Poor appetite | ACS |
|  | Raised creatinine | Moore et al, 2021 |
|  | Raised white cell count | Moore et al, 2021 |
|  | Swelling in the feet | ACS |
|  | Urinary tract infection | Koo et al, 2018 |
|  | Visible hematuria | Koo et al, 2018 |
|  | Weight loss | ACS |
| Colorectal | Abdominal bloating | Koo et al, 2018 |
|  | Abdominal pain | Moore et al, 2021 |
|  | Abdominal tenderness | Moore et al, 2021 |
|  | Anemia | Moore et al, 2021 |
|  | Loss of appetite | Moore et al, 2021 |
|  | Back pain | Koo et al, 2018 |
|  | Blood in stool | ACS/Rasmussen et al, 2019 |
|  | Change in bowel habit | Moore et al, 2021 |
|  | Constipation | Moore et al, 2021 |
|  | Diarrhea | Moore et al, 2021 |
|  | Iron deficiency | Moore et al, 2021 |
|  | Rectal bleeding | Moore et al, 2021 |
|  | Weakness and fatigue | ACS/Rasmussen et al, 2019 |
|  | Weight loss | Moore et al, 2021 |
| Esophagus | Abdominal pain | Koo et al, 2018 |
|  | Abdominal tenderness | Koo et al, 2018 |
|  | Anemia | Koo et al, 2018 |
|  | Chronic cough | ACS |
|  | Constipation | Moore et al, 2021 |
|  | Dyspepsia | Koo et al, 2018 |
|  | Dysphagia | Koo et al, 2018 |
|  | GI bleed | Koo et al, 2018 |
|  | Hematemesis | Koo et al, 2018 |
|  | Hoarseness | ACS |
|  | Low hemoglobin | Moore et al, 2021 |
|  | Nausea | Koo et al, 2018 |
|  | Poor appetite | Koo et al, 2018 |
|  | Reflux | Koo et al, 2018 |
|  | Thrombocytosis | Moore et al, 2021 |
|  | Vomiting | Koo et al, 2018 |
|  | Weight loss | Koo et al, 2018 |
| Kidney | Abdominal pain | Koo et al, 2018 |
|  | Abdominal tenderness | Koo et al, 2018 |
|  | Anemia | Koo et al, 2018 |
|  | Back pain | Koo et al, 2018 |
|  | Constipation | Koo et al, 2018 |
|  | Deep vein thrombocytosis | Koo et al, 2018 |
|  | Fever | ACS |
|  | Invisible hematuria | Koo et al, 2018 |
|  | Malaise | Koo et al, 2018 |
|  | Microcytosis | Moore et al, 2021 |
|  | Nausea | Koo et al, 2018 |
|  | Poor appetite | Koo et al, 2018 |
|  | Thrombocytosis | Moore et al, 2021 |
|  | Urinary tract infection | Koo et al, 2018 |
|  | Visible hematuria | Koo et al, 2018 |
|  | Weight loss | Koo et al, 2018 |
| Leukemia | Abdominal pain | Koo et al, 2018 |
|  | Abdominal tenderness | Koo et al, 2018 |
|  | Bleeding gums | Koo et al, 2018 |
|  | Breathlessness | Koo et al, 2018 |
|  | Bruises | ACS |
|  | Chest pain | Koo et al, 2018 |
|  | Chronic cough | Koo et al, 2018 |
|  | Diarrhea | Koo et al, 2018 |
|  | Dizzy | ACS |
|  | Fever | Koo et al, 2018 |
|  | Flu | Koo et al, 2018 |
|  | Heavy menstruation | ACS |
|  | Hypertension | Koo et al, 2018 |
|  | Malaise | Koo et al, 2018 |
|  | Nausea | Koo et al, 2018 |
|  | Nosebleeds | Koo et al, 2018 |
|  | Pale skin | ACS |
|  | Vomiting | Koo et al, 2018 |
|  | Weight loss | Koo et al, 2018 |
| Liver | Abdominal bloating | ACS |
|  | Abdominal pain | ACS |
|  | Abdominal tenderness | ACS |
|  | Appetite loss | ACS |
|  | Enlarged liver | ACS |
|  | Enlarged spleen | ACS |
|  | Fluid build up in abdomen | ACS |
|  | Itching | ACS |
|  | Jaundice | ACS |
|  | Nausea | ACS |
|  | Vomiting | ACS |
|  | Weight loss | ACS |
| Lung | Abnormal spirometry | Koo et al, 2018 |
|  | Breathlessness | Koo et al, 2018 |
|  | Chest pain | Koo et al, 2018 |
|  | Chronic cough | Koo et al, 2018 |
|  | Finger clubbing | Koo et al, 2018 |
|  | Hemoptysis | Koo et al, 2018 |
|  | Hoarseness | Koo et al, 2018 |
|  | Lymphadenopathy | Koo et al, 2018 |
|  | Malaise | Koo et al, 2018 |
|  | Poor appetite | Koo et al, 2018 |
|  | Recurrent chest infection | Moore et al, 2021 |
|  | Shoulder pain | Koo et al, 2018 |
|  | Stridor | Koo et al, 2018 |
|  | Superior vena cava obstruction | Koo et al, 2018 |
|  | Thrombocytosis | Koo et al, 2018 |
|  | Weight loss | Koo et al, 2018 |
| Lymphoma | Abdominal bloating | ACS |
|  | Abdominal pain | Koo et al, 2018 |
|  | Abdominal tenderness | Koo et al, 2018 |
|  | Back pain | Koo et al, 2018 |
|  | Breathlessness | Koo et al, 2018 |
|  | Bruises | ACS |
|  | Chills | ACS |
|  | Constipation | Koo et al, 2018 |
|  | Dyspepsia | Koo et al, 2018 |
|  | Lymphadenopathy | Koo et al, 2018 |
|  | Malaise | Koo et al, 2018 |
|  | Nausea | Koo et al, 2018 |
|  | Vomiting | Koo et al, 2018 |
|  | Weight loss | Koo et al, 2018 |
| Myeloma | Anemia | ACS |
|  | Back pain | Koo et al, 2018 |
|  | Bone pain | Koo et al, 2018 |
|  | Bone weakness | ACS |
|  | Breathlessness | Koo et al, 2018 |
|  | Chest pain | Koo et al, 2018 |
|  | Fracture | Koo et al, 2018 |
|  | Hypercalcemia | ACS |
|  | Hyperviscosity | ACS |
|  | Joint pain | Koo et al, 2018 |
|  | Leukopenia | ACS |
|  | Nausea | Koo et al, 2018 |
|  | Nosebleeds | Koo et al, 2018 |
|  | Peripheral neuropathy | ACS |
|  | Recurrent chest infection | Koo et al, 2018 |
|  | Spinal cord compression | ACS |
|  | Thrombocytopenia | ACS |
|  | Weight loss | Koo et al, 2018 |
| Pancreas | Abdominal pain | Moore et al, 2021 |
|  | Abdominal tenderness | Moore et al, 2021 |
|  | Back pain | Moore et al, 2021 |
|  | Constipation | Moore et al, 2021 |
|  | Dark urine | ACS |
|  | Deep vein thrombosis | ACS |
|  | Diarrhea | Moore et al, 2021 |
|  | Gallbladder enlargement | ACS |
|  | Itchy skin | ACS |
|  | Jaundice | Stapley et al, 2012 & Moore et al, 2021 |
|  | Light colored or greasy stools | ACS |
|  | Liver enlargement | ACS |
|  | Malaise | Moore et al, 2021 |
|  | Nausea | Moore et al, 2021 |
|  | Poor appetite | ACS |
|  | Pulmonary embolism | ACS & Moore et al, 2021 |
|  | Vomiting | Moore et al, 2021 |
|  | Weight loss | Stapley et al, 2012 |
| Stomach | Abdominal bloating | ACS |
|  | Abdominal pain | Moore et al, 2021 & Rasmussen et al, 2018 |
|  | Abdominal tenderness | Moore et al, 2021 & Rasmussen et al, 2018 |
|  | Anemia | Moore et al, 2021 |
|  | Appetite loss | ACS & Rasmussen et al, 2018 |
|  | Blood in stool | ACS |
|  | Chest pain | Moore et al, 2021 |
|  | Constipation | Moore et al, 2021 |
|  | Dyspepsia | Moore et al, 2021 |
|  | Dysphagia | Moore et al, 2021 & Rasmussen et al, 2018 |
|  | Fluid buildup in abdomen | ACS |
|  | Hematemesis | Moore et al, 2021 |
|  | Low hemoglobin | Moore et al, 2021 |
|  | Nausea | Moore et al, 2021 & Rasmussen et al, 2018 |
|  | Reflux | Moore et al, 2021 |
|  | Thrombocytosis | Moore et al, 2021 |
|  | Vomiting | Moore et al, 2021 & Rasmussen et al, 2018 |
|  | Weakness and fatigue | ACS |
|  | Weight loss | Moore et al, 2021 & Rasmussen et al, 2018 |
| Ovary | Abdominal bloating | Koo et al, 2018 |
|  | Abdominal distension | Koo et al, 2018 |
|  | Abdominal pain | Koo et al, 2018 |
|  | Abdominal tenderness | Koo et al, 2018 |
|  | Back pain | Koo et al, 2018 |
|  | Blood in stool | Koo et al, 2018 |
|  | Change in bowel habit | Koo et al, 2018 |
|  | Constipation | Koo et al, 2018 |
|  | Diarrhea | Koo et al, 2018 |
|  | Fluid buildup in abdomen | Moore et al, 2021 |
|  | Increased frequency of urination | Koo et al, 2018 |
|  | Irregular menses | Koo et al, 2018 |
|  | Lump in abdomen | Koo et al, 2018 |
|  | Nausea | Koo et al, 2018 |
|  | Poor appetite | Koo et al, 2018 |
|  | Postmenopausal bleeding | Koo et al, 2018 |
|  | Rectal bleeding | Koo et al, 2018 |
|  | Reflux | Koo et al, 2018 |
|  | Vomiting | Koo et al, 2018 |
|  | Weakness and fatigue | Koo et al, 2018 |
|  | Weight loss | Koo et al, 2018 |
| Uterus | Abdominal pain | Koo et al, 2018 |
|  | Abdominal tenderness | Koo et al, 2018 |
|  | High blood glucose | Moore et al, 2021 |
|  | Invisible hematuria | Koo et al, 2018 |
|  | Irregular menses | Koo et al, 2018 |
|  | Low hemoglobin | Moore et al, 2021 |
|  | Thrombocytosis | Moore et al, 2021 |
|  | Visible hematuria | Koo et al, 2018 |

* Koo MM, Swann R, McPhail S, Abel GA, Elliss-Brookes L, Rubin GP, Lyratzopoulos G. Presenting symptoms of cancer and stage at diagnosis: evidence from a cross-sectional, population-based study. Lancet Oncol. 2020 Jan;21(1):73-79. doi: 10.1016/S1470-2045(19)30595-9. Epub 2019 Nov 6. PMID: 31704137; PMCID: PMC6941215.

Moore SF, Price SJ, Chowienczyk S, Bostock J, Hamilton W. The impact of changing risk thresholds on the number of people in England eligible for urgent investigation for possible cancer: an observational cross-sectional study. Br J Cancer. 2021 Nov;125(11):1593-1597. doi: 10.1038/s41416-021-01541-4. Epub 2021 Sep 16. PMID: 34531548; PMCID: PMC8445014.

Signs and Symptoms of Cancer. American Cancer Society. Accessed May 13, 2025. https://www.cancer.org/cancer/diagnosis-staging/signs-and-symptoms-of-cancer.html

Rasmussen S, Haastrup PF, Balasubramaniam K, Elnegaard S, Christensen RD, Storsveen MM, Søndergaard J, Jarbøl DE. Predictive values of colorectal cancer alarm symptoms in the general population: a nationwide cohort study. Br J Cancer. 2019 Mar;120(6):595-600. doi: 10.1038/s41416-019-0385-x. Epub 2019 Feb 22. PMID: 30792531; PMCID: PMC6461905.

Stapley S, Peters TJ, Neal RD, Rose PW, Walter FM, Hamilton W. The risk of pancreatic cancer in symptomatic patients in primary care: a large case-control study using electronic records. Br J Cancer. 2012 Jun 5;106(12):1940-4. doi: 10.1038/bjc.2012.190. Epub 2012 May 22. PMID: 22617126; PMCID: PMC3388562.
